# Clinical trial to analyze the effects of oral intake of *Phellinus linteus* (sanghuang) extract on immune function: a study protocol for a randomized, double-blind controlled trial

**DOI:** 10.1186/s13063-021-05740-5

**Published:** 2021-11-27

**Authors:** Yong Ho Ku, Hyun Lee, Hwa Yeon Ryu, Jae Hui Kang

**Affiliations:** 1 Department of Acupuncture & Moxibustion Medicine, College of Korean Medicine, Daejeon University, Daejeon, Republic of Korea; 2 Department of Acupuncture & Moxibustion Medicine, Cheonan Korean Medicine Hospital of Daejeon University, Cheonan-si, Republic of Korea

**Keywords:** Immune function, *Phellinus linteus*, Randomized controlled trial

## Abstract

**Background:**

As the population of Korea ages, interest in healthcare has increased. In particular, there is an increasing demand for immune-function improvement to prevent infectious diseases. *Phellinus linteus* (PL) has previously been shown to exert immune-enhancing and anticancer effects. We aim to evaluate whether PL mycelium extract, cultured from the PL KCTC0399BP strain, can increase immune function, as measured using blood-test indicators. This clinical trial protocol is designed as the main trial and is based on the results of a pilot study.

**Methods:**

This clinical trial is a randomized, double-blinded, placebo-controlled trial. Ninety-eight participants are enrolled and randomly divided into two groups: the experimental group (PL 1000 mg) and the control group (placebo). Participants are administered with experimental food or placebo for eight weeks. Blood tests are performed before trial initiation and 8 weeks later, at trial completion. Laboratory evaluation items are as follows: natural killer cell activity, tumor necrosis factor-α, interferon-γ, interleukin (IL)-1β, IL-2, IL-6, IL-12, immunoglobulin (Ig)G1, IgG2, and IgM. We will mainly use the full analysis dataset to statistically analyze the effectiveness of the treatment.

**Discussion:**

This study evaluates the effects of PL extract on immune function and will contribute to knowledge on the value of PL as an immune-function–boosting functional food.

**Trial registration:**

Clinical Research Information Service (CRIS) of Korea CRIS-KCT0005460. Registered on 12 October 2020

## Introduction

There is global, growing interest in human health, and the demand for medicines and foods that boost immune function is increasing with the advent of aging populations and the spread of infectious diseases [1]. Immunity is a means of defense that protects our bodies from external influences. From mild diseases such as colds to infectious diseases and cancer, immunity is a key factor.

*Phellinus linteus* (PL) has established immune-improving functions, and it has been reported to increase the activity of interleukin (IL)-12, interferon (IFN)-γ, and natural killer (NK) cells [2, 3]. In previous studies, it has been reported that PL extract increases immunity, mediated by cells such as T lymphocytes, NK cells, macrophages, and B lymphocytes [4], and exhibits antitumor and antioxidant activities [5].

In a patent for an extract produced using the existing PL KCCM KSSW01 strain, the immunity-enhancing effect of the glucomannan in the extract was proven [6]. Glucomannan consists of 53.7% glucose, 23.5% mannose, 7.7% galactose, 7.1% xylose, 5.8% fructose, and 2.2% 3-O-methylglucose. The PL extract used in the present study, which was produced using the PL KCTC0399BP strain, contains galactomannoglucan. Galactomannoglucan comprises 78.6% glucose, 18.0% mannose, and 3.4% galactose and is thus quite different from glucomannan [7].

The PL extract used in this clinical trial is marketed as Mesima, an immunostimulant, in the Republic of Korea. This clinical trial aims to analyze and evaluate whether this PL extract, when used as an ingredient of a functional health food, can effectively improve immune function. We evaluate changes in (1) NK cell activity and (2) cytokines (white blood cells [WBC]; tumor necrosis factor [TNF]-α; IFN-γ; immunoglobulin [Ig]G1, IgG2, and IgM; and IL-1β, IL-2, IL-6, and IL-12) to verify the efficacy of the PL extract in preventing immunity weakening. This clinical trial is the main trial, and it is designed to confirm efficacy and stability based on the results of a pilot study.

## Methods

### Study design

This is a prospective, double-blind, single-center, randomized, controlled clinical trial designed to demonstrate the effects of PL extract on immune function. Recruitment starts in October 2020 and the trial ends in March 2021. Subjects are recruited via placards on outdoor billboards and banners in Daejeon University Cheonan Korean Medicine Hospital. All subjects receive written explanations and informed consent forms for the clinical trial protocol from a Korean medical doctor (KMD). The KMD also comprehensively explains the study to the recruited subjects, receives informed consent from them, and selects participants in the study based on detailed screening.

The study planned to recruit 98 subjects who will be randomly assigned in a 1:1 ratio to either the experimental group (PL 1000 mg) or the control group (placebo). The subjects take one 500 mg capsule twice daily for eight weeks, and this capsule contains either PL or dextrin, depending on the group they had been assigned to.

**Fig. 1:**
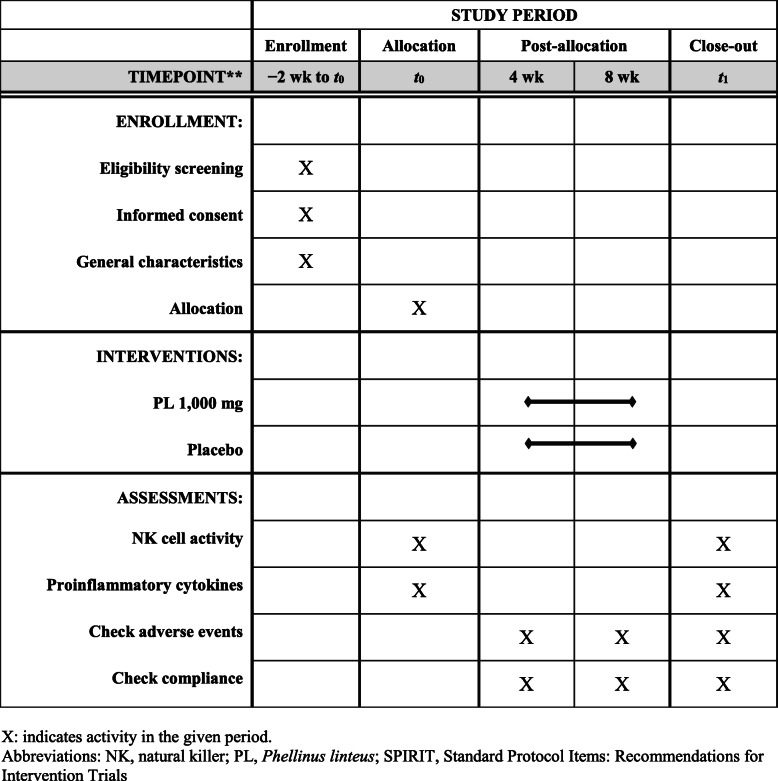
SPIRIT checklist showing the schedule of enrollment, assessments, and allocation for each subject in the *Phellinus linteus* extract clinical trial protocol

The variables we evaluated are NK cell activity, TNF-α, IFN-γ, IL-1β, IL-2, IL-6, IL-12, IgG1, IgG2, and IgM. Blood samples are taken for this laboratory test on the first day of participation, before the subjects has begun ingesting the test food, and 8 weeks later, at the end of the trial. The subjects visit the clinic three times and the clinical trial span a total of eight weeks (Table 1, Fig. 1).

**Table 1:** Clinical trial process for the *Phellinus linteus* extract clinical trial protocol

Section	Screening	Visit 1 (baseline visit)	Visit 2 (during trial)	Visit 3 (trial end)
	(− 2 weeks to 0 days)	(0 days)	(4 weeks ± 5 days)	(8 weeks ± 5 days)
Prepare a written consent form	√			
Evaluate inclusion/exclusion criteria	√			
Examine subject information	√			
Assign screening number	√			
Assign random numbers to subjects		√		
Investigate medical history and medication history	√	√		
Conduct physical examination	√	√	√	√
Assess vital signs	√	√	√	√
Conduct clinical laboratory blood test	√	√		√
Conduct pregnancy test	√			
Observe upper respiratory tract infection symptoms		√	√	√
Distribute experimental/control foods		√	√	
Measure effectiveness-evaluation variables		√		√
Check adverse events			√	√
Identify concomitant drugs			√	√
Check compliance			√	√
Confirm cancelation and withdrawal criteria		√	√	√

### Inclusion and exclusion criteria

The inclusion criteria are as follows: (1) age of 20–65 years, (2) peripheral white blood cell count of 3–10 × 10^3^/μL, (3) upper respiratory tract infection or symptoms of a common cold at least twice within the year preceding the beginning of the trial, and (4) agreement to participate in this trial and voluntary signing of a written informed consent form (by the subject or a legal representative).

The exclusion criteria are as follows: (1) currently being treated for a clinically significant acute or chronic cardiocerebrovascular, immune, respiratory, hepatobiliary, kidney, urinary, nervous, musculoskeletal, psychotic, infectious, or hematologic/neoplastic disease (however, it was possible to participate in the study at the discretion of investigator who evaluated the subjects); (2) uncontrolled hypertension (160/100 mmHg or more, measured after 10 min of rest); (3) uncontrolled diabetes (fasting blood glucose ≥ 126 mg/dL, or commencement of a new drug to treat diabetes within the previous 3 months); (4) aspartate aminotransferase (AST) (glutamic-oxaloacetic transaminase [GOT]) or alanine aminotransferase (ALT) (glutamic-pyruvic transaminase [GPT]) blood level ≥ 3 × the normal upper limit of the institution; (5) creatinine blood levels > 2.4 mg/dL in males and > 1.8 mg/dL in females; (6) ingestion of nutritional supplements, which can affect immunity, within 2 weeks before the screening; (7) severe gastrointestinal symptoms, such as heartburn or indigestion; (8) pregnancy, lactation, or an intention to fall pregnant during the study period; (9) sensitivity or allergy to foods related to the test food; (10) concurrent participation in other studies; (11) ingestion of other experimental drugs within four weeks of starting this study; and (12) unsuitability for this study (as determined by the investigator) based on overall health status.

### Dosage calculation

A dose of 200 mg/kg administered to mice for 1 week did not exhibit any specific toxicity. This effective dose was converted to a human intake dose of 960 mg/d (16 mg/kg/day), based on an adult body weight of 60 kg and a conversion factor of 0.08, which takes the relationship of body weight to body surface area into account. In a preliminary clinical study in which PL extract was ingested at 1000 or 2000 mg/day, no related adverse reactions were observed, and it was confirmed that immune function improved at 1000 mg/day. Therefore, the PL extract intake is set to 1000 mg/day for this study, based on the results of these preliminary clinical and animal studies.

### Sample size calculation

The necessary sample size is calculated using the NK cell activity results from the pilot study, in which this variable is used to evaluate efficacy. Calculations are performed using G*power 3.1.9.4, and the minimum number of subjects required to confirm statistical significance is determined to be 41 individuals per group. Accounting for a dropout rate of 15%, we aim to recruit a total of 98 people, with 49 for each group.

### Randomization and blinding procedures

The final registered subjects are assigned in a 1:1 ratio to the experimental and control groups using block randomization. The size of the blocks is set as random by statisticians. The investigators are therefore blinded to the size and number of blocks used for the random assignments. The total number of random assignments is generated on a scale of approximately 120% of the target recruitment number. A three-digit randomized number is assigned to each subject and the test food or a placebo is distributed to the subjects according to this number. Both the investigators and the subjects are blinded to the subjects’ treatment group. Randomization details are generated by statisticians using nQuery Advisor 7.0 (or SAS 9.0 or SPSS 21.0). Each subject’s randomized number and group-assignment details are placed in a nonpermeable, sealed bag, which was given to the supervisor for storage and management. Once a randomized number has been assigned, it is not reused, even if the subject it is first assigned to dropped out of the study.

### Intervention

**Fig. 2:**
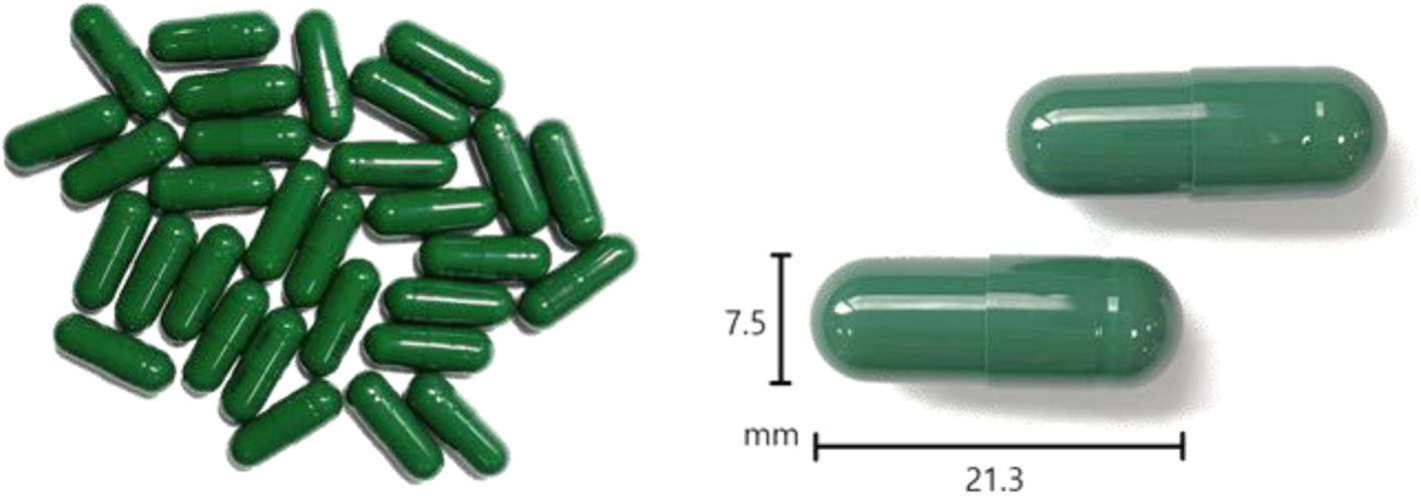
*Phellinus linteus* extract and placebo capsules, showing their identical appearance

The test food is obtained from Hankookshinyak Pharmaceutical (Nonsan, Republic of Korea). The appearance and physical properties of the test food and placebo are identical: no difference could be observed with the naked eye and the weight is identical (Table 2, Fig. 2). The placebo contains 500 mg dextrin per capsule, and the test food contains the same quantity of the PL extract. In addition, the capsules are placed in identically labeled paper envelopes, to ensure that the researchers and subjects remained blinded to treatment assignments.

**Table 2:** Ingredients of *Phellinus linteus* extract

Ingredient	Quantity/percentage
Calories (Kcal/100 g)	346.36 Kcal/100 g
Carbohydrate (%)	68.86%
Crude protein (%)	15.75%
Crude fat (%)	0.88%
Sodium (mg/100 g)	71.57 mg/100 g
Sugars (mg/g)	128.29 mg/g
Saturated fat (g/100 g)	0.07 g/100 g
Trans fat (g/100 g)	–
Cholesterol (mg/100 g)	–

The subjects ingest one capsule twice a day, 30 min after meals, for 8 weeks. The investigator collects the empty envelopes after every visit by the subjects, to verify that they had taken their treatment. Any other treatment received during their participation in the trial, functional health foods taken, or additional therapies are considered to be combination therapy. If coadministration is necessary, the administration details are recorded in the case report form (CRF) and treatment chart, and the existing therapy remained unchanged. Concomitant drug administration was minimized during trial participation, however. Concomitant drug administration is allowed based on the judgment of principal investigator, when it is deemed inconsequential to the trial. If a drug directly affected immune function, that subject withdraws from the trial.

### Outcome measures

The primary outcome is the amount of change in NK cell activity. NK cells play a key role in immune function against viruses and have recently been shown to play an important role in anticancer activity [8]. NK cell activity is measured to evaluate whether immune function increased as a result of the treatment. The secondary outcome is the amount of change in WBC in the peripheral blood and in the levels of TNF-α, IFN-γ, IL-1β, IL-2, IL-6, IL-12, IgG1, IgG2, and IgM. Proinflammatory cytokines such as TNF-α, IFN-γ, IL-1β, IL-2, IL-6, and IL-12 are factors that regulate the inflammatory responses of tissues [9]. Immunoglobulins such as IgG1, IgG2, and IgM are involved in virus neutralization and aggregation reactions. They also play a major role in immunity against influenza [10]. Changes in immune function can thus be confirmed by measuring changes in cytokines and immunoglobulins.

### Data collection and monitoring

Outcome measures are taken before first administering the test food (visit 1) and after 8 weeks, at the end of administration (visit 3), via laboratory tests. For the screening, AST, ALT, ALP, γ-GTP, total cholesterol, FBS, total bilirubin, BUN, creatinine, CRP, ESR, WBC, RBC, Hb, Hct, platelets, triglyceride, HDL, LDL, Na, K, and Cl are measured. At visit 1, NK cell activity, TNF-α, IFN-γ, IL-1β, IL-2, IL-6, IL-12, IgG1, IgG2, and IgM are also measured, and AST, ALT, ALP, γ-GTP, total cholesterol, FBS, total bilirubin, BUN, creatinine, CRP, ESR, WBC, RBC, Hb, Hct, platelets, triglyceride, HDL, LDL, Na, K, Cl, NK cell activity, TNF-α, IFN-γ, IL-1β, IL-2, IL-6, IL-12, IgG1, IgG2, and IgM are measured at visit 3. Personal information is collected during screening. The investigator processes the data, including personal information, under the clinical study protocol approved by the IRB. The subjects’ names remain confidential and the subjects are identified only according to their assigned randomized number. The principal investigator and sub-investigator secure the data, including personal information, in locked cabinets. Access to the data is restricted. All data are handed to storage manager after the clinical trial results have been reported, and the data is preserved for 3 years from the date of completion of the trial. When requested by monitors, inspection implementers, the IRB, or the Minister of Food and Drug Safety, the relevant documents are provided for access. The principal investigator requests the contract research organization (CRO) to monitor the study according to the plan and schedule. The CRO is independent from the sponsor and any competing interests. The CRO reviews the consent forms and evidence documentation and checked the capture of the main evidence data for quality control. If necessary, the CRO may request an investigator interview. Monitoring is conducted at the beginning of, during, and at the end of the trial by LAB to MEDI CRO (Seoul, Republic of Korea). During monitoring, research plans, case records, and annexed documents are reviewed to check whether they had been scientifically performed according to the plans approved by the IRB, and to ensure that the safety and rights of the research subjects are not infringed.

### Statistical analysis

Demographic data collected during screening will be tested using *t* tests (or Wilcoxon rank–sum tests) for continuous data. For categorical data, a chi-squared test (or Fisher’s exact test) will be performed. Effectiveness evaluation will be based on the results of the full analysis dataset. The per-protocol (PP) group will also be analyzed for reference. Statistical significance will be determined based on a significance level of 5%. If the difference between the two groups is normally distributed, an independent *t* test will be performed; if not, a Mann–Whitney *U* test will be performed. To test for differences before and after the treatment within a group, paired *t* tests will be used for normally distributed data; otherwise, Wilcoxon’s signed-rank tests will be used. The Shapiro–Wilk test will be performed to test the normality of continuous variables. When a difference between groups is observed, the drinking history variable will be considered for control variable, and additional covariate analysis will be performed. In the full dataset analysis, missing values will be replaced by the last observation carried forward. For the safety evaluation, the 95% confidence interval for each group will be determined by calculating the rate of occurrence of adverse reactions, and comparisons will be made between the groups. From the results of each laboratory test, continuous data such as vital signs will be compared with the baseline and analyzed using either a *t* test or a paired *t* test. If the assumption of normality is not satisfied, the Wilcoxon signed-rank test or the Wilcoxon rank–sum test will be performed. Categorical data will be analyzed using chi-squared tests and Fisher’s exact tests to determine whether there are differences between the groups in terms of the frequency and ratio of each category.

### Withdrawal and dropout

Subjects who have taken the test food as part of the clinical trial for 8 weeks are considered to have completed the trial. Subjects are excluded from the trial in the following cases: violation of the inclusion/exclusion criteria (during screening only), request to withdraw made by the subject, occurrence of a serious adverse reaction or an adverse reaction that made continuation difficult, use of prohibited drugs that may have affected the efficacy-evaluation variables, loss of contact with the subject, consumption of medicines or functional health foods that may have affected the outcome, pregnancy during the trial period, and determination by the investigators.

All causes for dropping out of the study are documented in detail in the CRF. A laboratory test is also performed to assess the safety of subjects who dropped out. If an adverse event (AE) has occurred, follow-up observations are conducted and reported until the cause had been identified. In the case of serious adverse reactions, the IRB is notified immediately.

### Safety

Side effects, such as AEs, adverse drug reactions, and serious adverse events (SAEs), are checked at visits 2 and 3. All AEs are classified and evaluated as mild, moderate, or severe. If an SAE occurred, it is reported to the IRB within 24 h of identification. The IRB reviews reports and develops appropriate follow-up measures. The organization that implemented the clinical trial makes every effort to protect the subjects’ safety. The principal investigator stops the trial for any subject who experienced an SAE and takes appropriate measures, such as withdrawing the subject, and, if necessary, removing the blinding. A safety evaluation is conducted based on the rate of occurrence of side effects. Subjects are compensated for damages caused directly by the test food, in accordance with the policy. The institution running the trial has insurance to cover any harm caused by the trial. Subjects damaged directly as a result of participation in the clinical trial are provided with appropriate measures and compensation.

### Ethics

This clinical trial design is based on the Helsinki Declaration and the Korean Clinical Practice Guidelines. The protocol was approved by the IRB of the Daejeon University Cheonan Korean Medicine Hospital (DJUMC-2020-BM-07). When necessary, the protocol will be changed with permission from the IRB. The protocol was registered with the Clinical Research Information Service of the Republic of Korea (CRIS-KCT0005460).

## Discussion

The importance of immune function is increasing with the spread of infectious diseases in modern society worldwide. The immune system comprises various cytokines, immunoglobulins, and phagocytic cells that protect the body from external intruders. Infectious diseases related to immune systems are fatal in the elderly population, which has reduced immune function. Therefore, in modern societies with aging populations, the demand for foods that improve immune function is increasing.

PL extract has an anticancer effect because it improves immune function [11]. Studies have reported the anti-inflammatory effects of PL extract in rats [12], the anti-inflammatory mechanisms of PL extract in macrophages [13], and the antioxidant and antimicrobial activities of PL extract [14]. NK cells, which are responsible for innate immunity, play important anticancer [15] and antiviral [16] roles in immune function. According to the functional health food evaluation guide distributed by the Ministry of Food and Drug Safety in the Republic of Korea, NK cell activity should be evaluated by culturing effectors and target cells together, followed by flow cytometry or lactate dehydrogenase cytotoxicity assay kits. The lactate dehydrogenase assay is a method of measuring cell death that evaluates NK cell activity by analyzing NK cell cytotoxicity [17]. In existing clinical trials, a diagnostic kit is used to measure NK cell activity. The kit artificially activates NK cells using engineered recombinant cytokines, and then measures NK cell activity via IFN-γ detection. The kit has analysis limitations because it is not possible to evaluate NK cell activity using the effector to target concentration ratio. In this clinical trial, NK cell activity is confirmed using lactate dehydrogenase assays at various concentrations of effector to target cells (e.g., 12.5:1, 25:1, and 50:1). Changes in NK cell activity, proinflammatory cytokines, and Igs will be evaluated to confirm any improvement in immune function due to the consumption of PL extract. The study aims to verify the efficacy of PL extract as a health supplement by proving its beneficial effects on immune function.

## Data Availability

The data from this study will be provided upon reasonable request. The KMD participating in the clinical trials will have access to the final trial dataset. There are no contractual agreements that limit such access for investigators. The datasets for the completed study will be available for request following consent from the principal investigator.

## Abbreviations

ALT: Aminotransferase
AST: Aminotransferase
CRO: Contract research organization
GOT: Glutamic-oxaloacetic transaminase
GPT: Glutamic-pyruvic transaminase
PL: *Phellinus linteus*
NK: Natural killer
TNF: Tumor necrosis factor
IFN: Interferon
Ig: Immunoglobulin
IL: Interleukin
IRB: Institutional review board
